# The importance of input data on landslide susceptibility mapping

**DOI:** 10.1038/s41598-021-98830-y

**Published:** 2021-09-29

**Authors:** Krzysztof Gaidzik, María Teresa Ramírez-Herrera

**Affiliations:** 1 Institute of Earth Sciences, University of Silesia, Będzińska 60, 41-200 Sosnowiec, Poland; 2 Laboratorio de Tsunamis y Paleosismología, Instituto de Geografía, Universidad Nacional Autónoma de México, Ciudad Universitaria, 04510 Coyoacán, Ciudad de México, México

**Keywords:** Natural hazards, Solid Earth sciences, Geomorphology

## Abstract

Landslide detection and susceptibility mapping are crucial in risk management and urban planning. Constant advance in digital elevation models accuracy and availability, the prospect of automatic landslide detection, together with variable processing techniques, stress the need to assess the effect of differences in input data on the landslide susceptibility maps accuracy. The main goal of this study is to evaluate the influence of variations in input data on landslide susceptibility mapping using a logistic regression approach. We produced 32 models that differ in (1) type of landslide inventory (manual or automatic), (2) spatial resolution of the topographic input data, (3) number of landslide-causing factors, and (4) sampling technique. We showed that models based on automatic landslide inventory present comparable overall prediction accuracy as those produced using manually detected features. We also demonstrated that finer resolution of topographic data leads to more accurate and precise susceptibility models. The impact of the number of landslide-causing factors used for calculations appears to be important for lower resolution data. On the other hand, even the lower number of causative agents results in highly accurate susceptibility maps for the high-resolution topographic data. Our results also suggest that sampling from landslide masses is generally more befitting than sampling from the landslide mass center. We conclude that most of the produced landslide susceptibility models, even though variable, present reasonable overall prediction accuracy, suggesting that the most congruous input data and techniques need to be chosen depending on the data quality and purpose of the study.

## Introduction

Landslides are important landscape-forming factors that might impact human activities^1^. Damaging power of landslide material transported downslope can destroy human settlements, disrupt communications, break gas lines, water, and sewage, but most importantly, it could cause harm to people and the loss of life. The worldwide landslide database^2,3^ shows an average ca. 400 fatal landslides per year, which result in ~ 4,500 fatalities. The temporal occurrence of fatal landslides indicates a significant increasing trend in single-fatality landslides and those triggered by human activity^3^. Thus, landslide susceptibility mapping is a critical step in urban planning, land management, and safe human occupation, especially in mountainous areas of tropical climate frequently hit by hurricanes, where a significant majority of fatal landslides cluster.

Susceptibility to landslides can be mapped following numerous approaches^4^, grouped in (1) qualitative or heuristic^4–7^, and (2) quantitative methods^8–16^. The first ones are subjective methods based on expert's prior knowledge on the roles of geological and geomorphological factors on landslides. Quantitative approaches (deterministic, probabilistic, fuzzy logic, neural network, weight of evidence, logistic regression, etc.) are based on the integration of landslide-causing factors by using observed statistical relationships with landslides (probabilistic), machine learning, or by using numerical equations that explain physical mechanism of landsliding (i.e., deterministic).

The correctness of the landslide susceptibility model depends on the completeness and precision of landslide inventory, which highlights the distribution, types, and patterns of past landslides^17–19^, and the resolution of topographic data^20–23^, together with the number and quality of landslide causing-factors used for the modeling^24,25^. Landslide delineation can be made (1) manually using satellite images, aerial photographs, and field surveys^26–30^, and digital topographic data of various scales, up to centimeter-scale LIDAR (light detection and ranging) derived digital terrain models (DTM)^19,28,29,31^, or (2) automatically employing remote sensing techniques and computer algorithms^15,32,33^. Recently, automated methods of landslide identification using high-resolution LIDAR data have been particularly common^15,31–37^. The use of topographic data of different resolution and landslide inventories from different sources, together with various processing methods, rose questions: (1) how different would a susceptibility model be based on manual landslide inventory vs. a model based on automatically identified landslides? (2) How does the resolution of topographic data influence the resulting landslide susceptibility model? (3) Which landslide-causing factors are essential in each case to produce models with the highest accuracy, and overall, which model would be more accurate? (4) How does the sampling technique influence the accuracy of landslide susceptibility mapping?

**Figure 1:**
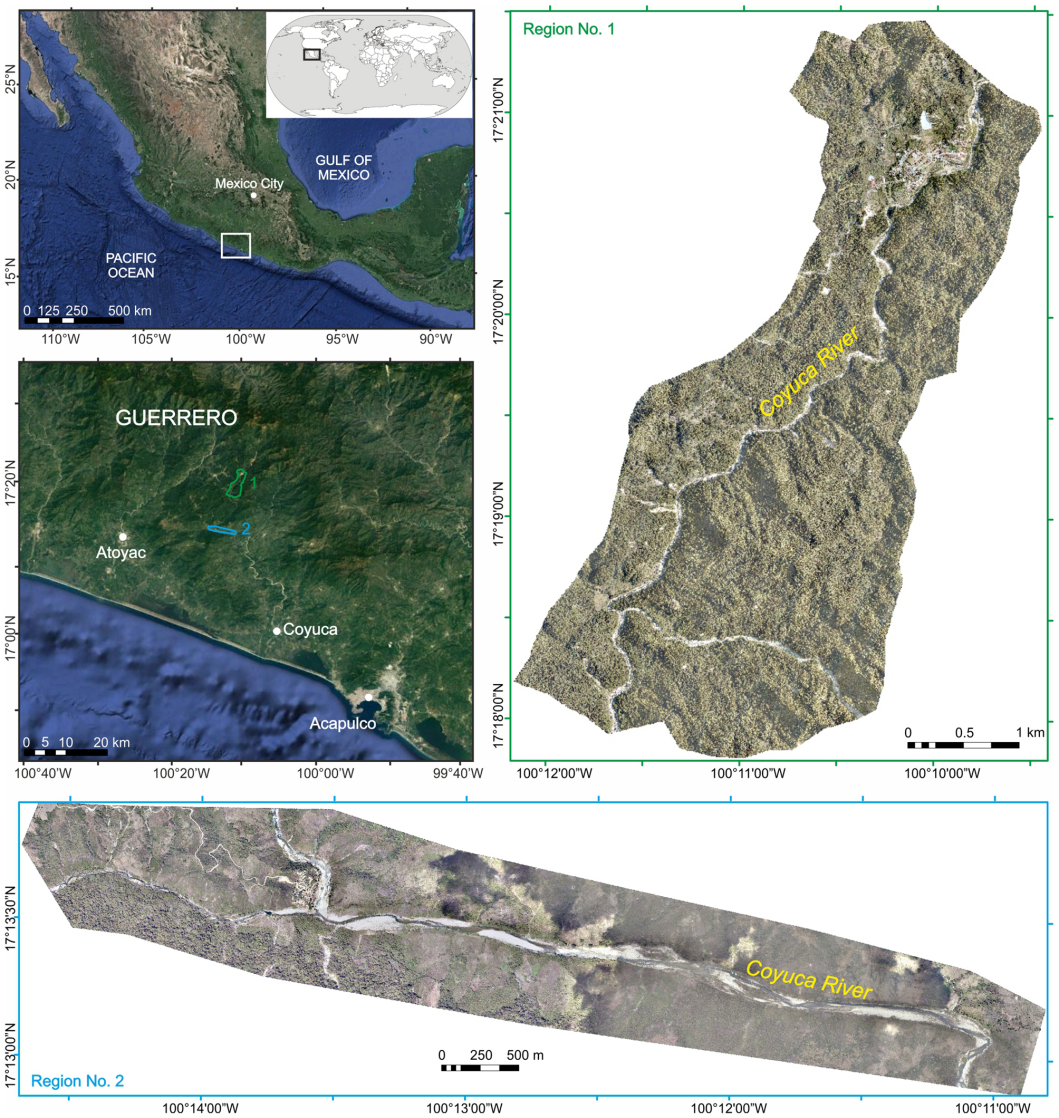
Orthophotomaps (10 cm resolution) of the studied regions, imposed on the shaded relief 1 m resolution LIDAR-derived digital terrain model (DTM), Coyuca drainage basin, southwest Mexico. Satellite images from Google Earth Pro 7.3 (Image © 2016 DigitalGlobe; https://www.google.com/intl/en/earth/). Maps were generated using ArcMap 10.2.2 software, copyright and licensed by ESRI (http://desktop.arcgis.com/en/). The final layout was created in ArcMap 10.2.2 (http://desktop.arcgis.com/en/) and CorelDRAW 2018 (https://www.coreldraw.com/en/).

Hence, the main goal of this study is to evaluate the influence of (1) the type of landslide inventory (manual or automatic), (2) spatial resolution of the topographic input data, (3) the number of landslide-causing factors, and (4) sampling techniques, on the accuracy of landslide susceptibility mapping. To answer these questions, we produced 32 landslide susceptibility models using the logistic regression method^15^ with different input data for two selected study regions in the Coyuca River basin in SW Mexico (Fig. 1). We discuss the impact of the variations in the input data on the precision and accuracy of the resulting models.

## Study area

To evaluate the effect of variations in input data on landslide susceptibility mapping, we chose two regions in the Coyuca River basin located within the Sierra Madre del Sur mountain range in Southwest Mexico (Fig. 1). This region has been recognized as highly susceptible to landslides because of frequent hurricanes, seismic activity, and anthropogenic land modifications^15^.

Selected regions cover an area of > 22 km^2^. The larger northern region (No. 1; 15.6 km^2^) encompasses upstream sections of the Coyuca River, characterized by relatively high local relief and steep slopes (Fig. 1 and Table 1). Dense tropical and subtropical forests dominate here (Fig. 1). The smaller southern region (No. 2; 6.7 km^2^) includes the E-W Coyuca River valley section, with asymmetric slopes, i.e., a steep south-facing slope covered with pastureland and cropland, and a gentler, densely vegetated north-facing slope. Poorly developed, very to extremely shallow, gravelly, or clayish soils, with little or no profile development on plutonic (granite and granodiorite) and metamorphic rocks (gneiss), dominate in both regions. Climate is humid to subhumid and warm^38^, with 1000–1100 mm/yr average annual precipitation rates. Rainfall is limited to five months of the rainy season (June to October), which is also the time of hurricanes that might trigger numerous landslides. Hurricane Manuel, a Category 1 hurricane (on the Saffir-Simpson Hurricane Wind Scale), made landfall as a tropical storm on the coast of Guerrero from the 13th to the 19th of September 2013 and resulted in 123 deaths and abundant landslide features in Southwest Mexico^39^.

**Table 1:** Area, elevation, slope, and lithology of studied regions (for location, see Fig. 1).

Parameter	Area (km^2^)	Elevation range (m amsl)	Mean elevation (m amsl)*	Mean slope (°)*	Lithology
Region					
No. 1	15.6	818.7–1,532.0	1,128.2 ± 124.6	29.5 ± 12.9	Granite, granodiorite
No. 2	6.7	318.5–653.0	426.3 ± 60.2	25.0 ± 12.4	Granite, granodiorite, gneiss

*Mean values with standard deviation.

## Results

### Landslide inventories

**Figure 2:**
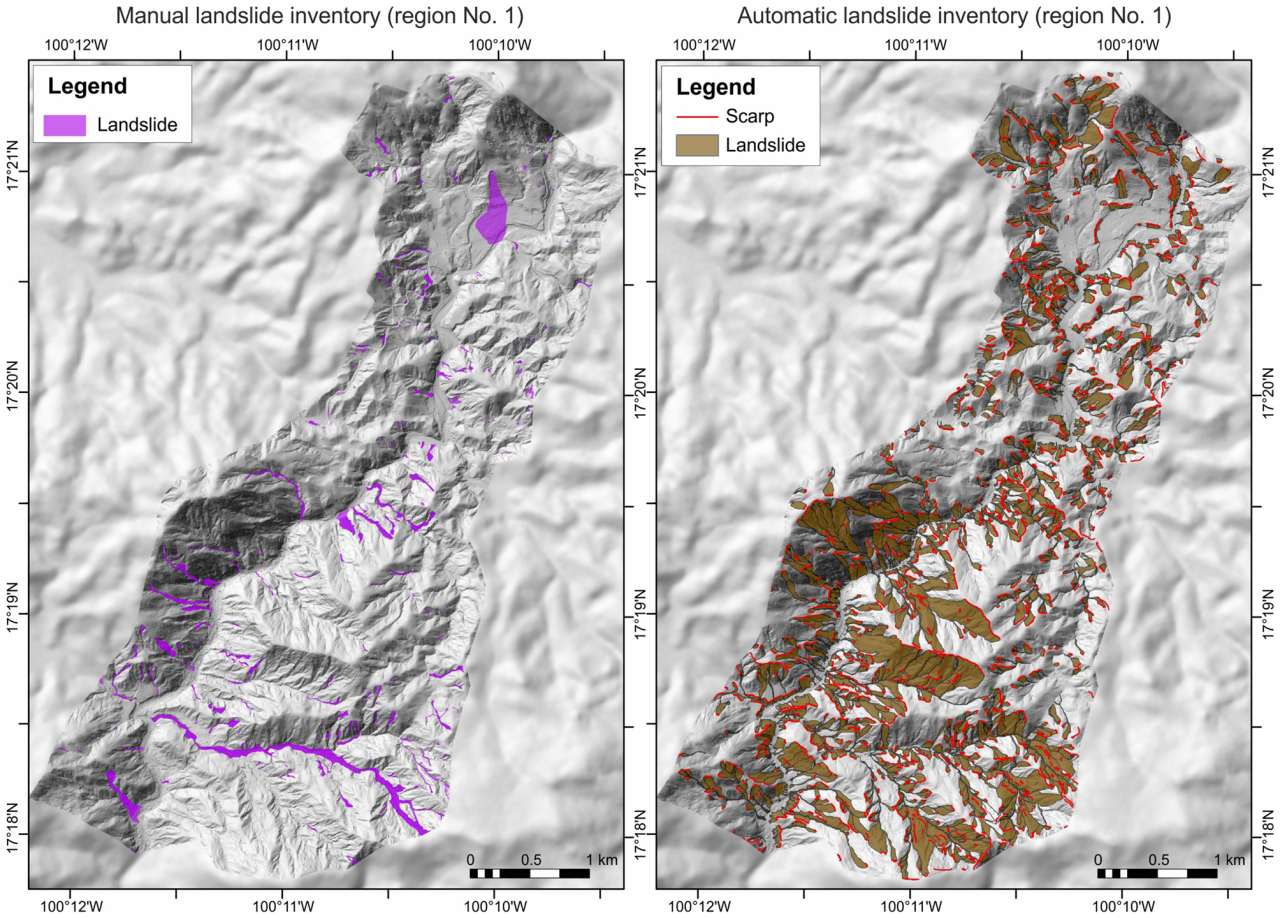
Landslides identified in region No. 1 using two approaches, including manual mapping using satellite images and automatic identification of landslide morphology employing CCM algorithm on shaded relief of LIDAR derived DTMs. Maps were generated using ArcMap 10.2.2 software, copyright and licensed by ESRI (http://desktop.arcgis.com/en/). The final layout was created in ArcMap 10.2.2 (http://desktop.arcgis.com/en/) and CorelDRAW 2018 (https://www.coreldraw.com/en/).

**Figure 3:**
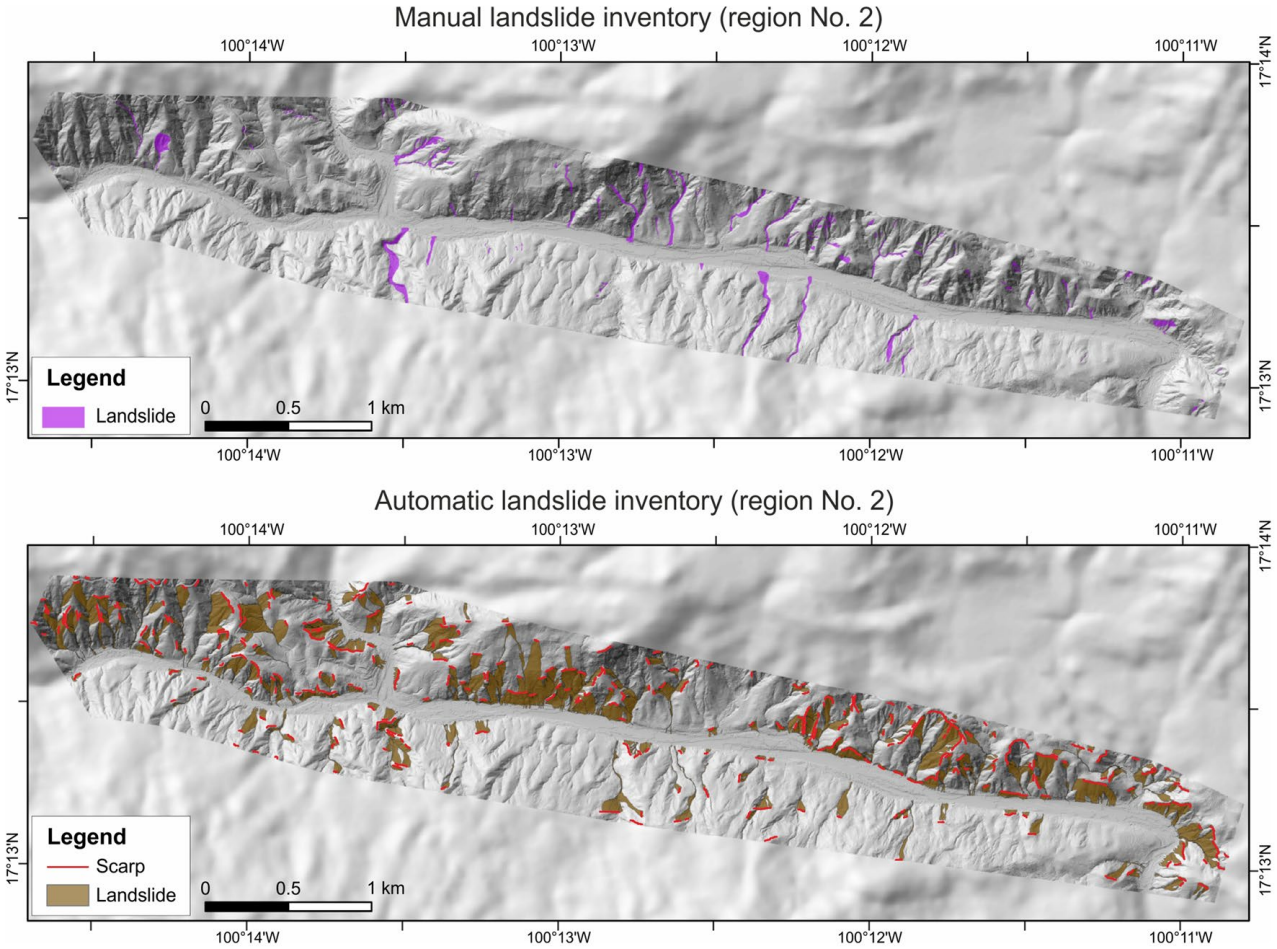
Landslides identified in region No. 2 using two approaches, including manual mapping using satellite images and automatic identification of landslide morphology employing CCM algorithm on shaded relief of LIDAR derived DTMs. Maps were generated using ArcMap 10.2.2 software, copyright and licensed by ESRI (http://desktop.arcgis.com/en/). The final layout was created in ArcMap 10.2.2 (http://desktop.arcgis.com/en/) and CorelDRAW 2018 (https://www.coreldraw.com/en/).

The manual inventory includes 419 landslides (241 in region No. 1, and 176 in region No. 2), represented mainly by small, narrow, and strongly elongated debris flows, i.e., average length ca. 100 m, width < 30 m and area < 1800 m^2^ ^15^. Only a few deep-seated landslides were observed, but these produced the largest damages, i.e., deep-seated La Pintada landslide that remobilized ca. 75,000 m^3^ of material and resulted in 71 fatalities destroying a large part of La Pintada Village^40^. The automatic method allowed us to identify four times more landslide features than manual identification, i.e., 1726. These are represented by debris flows and mudslides that occur after heavy precipitation, but also slumps and deep-seated landslides that do not fluidize and runout significantly^15^. The spatial distribution of landslides identified by both methods is presented in Figs. 2 and 3.

The comparison of the identified landslides with features verified in the field in a test area resulted in the very high values of overall accuracy, i.e., > 90% (99.67% for the manual inventory and 90.65%, for automatic inventory), comparable with literature data e.g.,^35,41^, proving their correctness. A lower value for automatic inventory is likely related to over-prediction of landslides e.g.,^15,32^. The reported over-estimation is a direct result of the primary objective of this method, i.e., capturing all past landslides. Consequently, some large rocks (especially in river bottoms), but also steep sections of rivers, and anthropogenic slopes might have been identified as landslide features.

### Landslide susceptibility models

**Figure 4:**
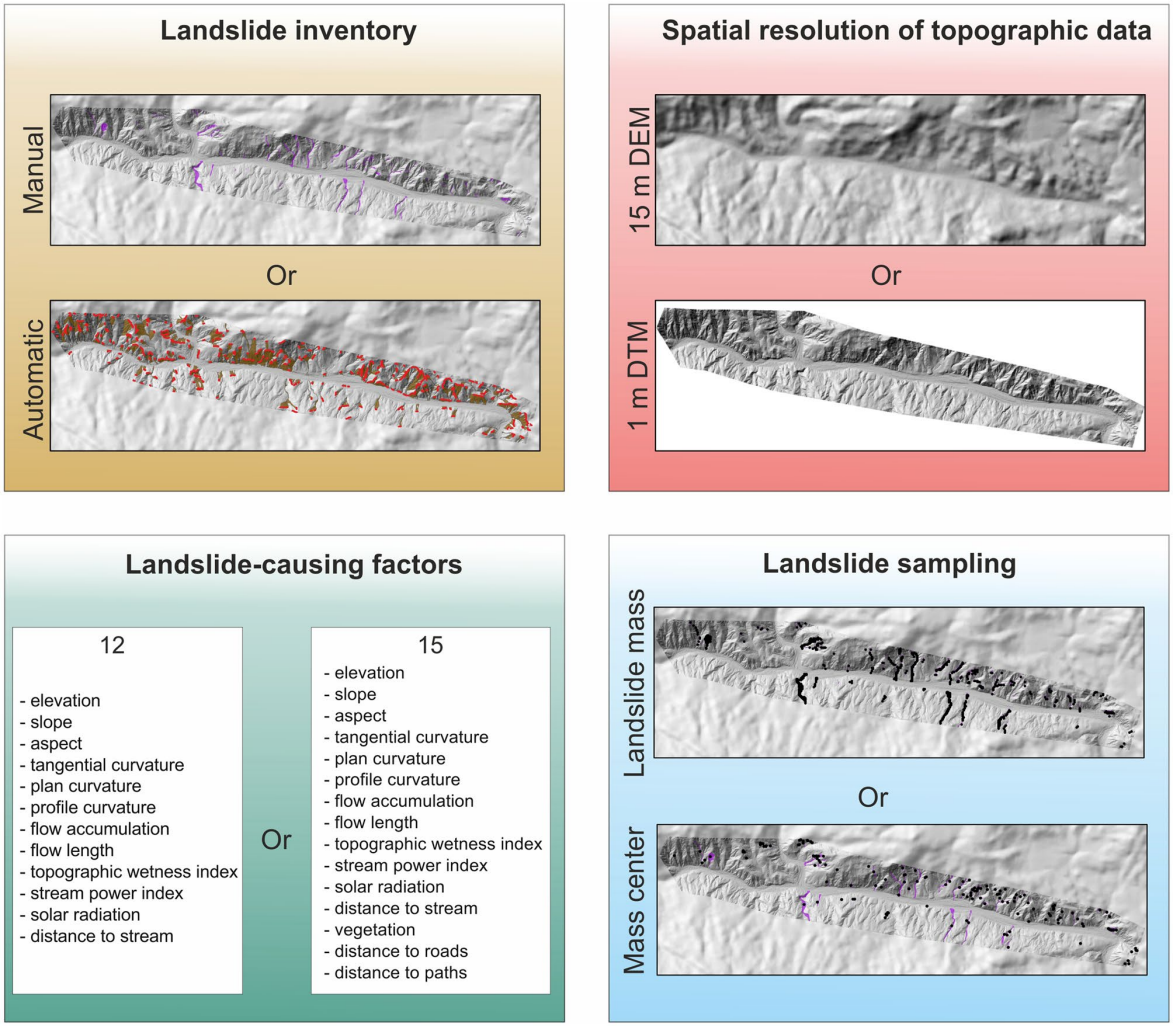
Differences in input data included in landslide susceptibility modeling using the logistic regression method for two selected study regions in the Coyuca River basin in SW Mexico. Maps were generated using ArcMap 10.2.2 software, copyright and licensed by ESRI (http://desktop.arcgis.com/en/). The final layout was created in CorelDRAW 2018 (https://www.coreldraw.com/en/).

**Figure 5:**
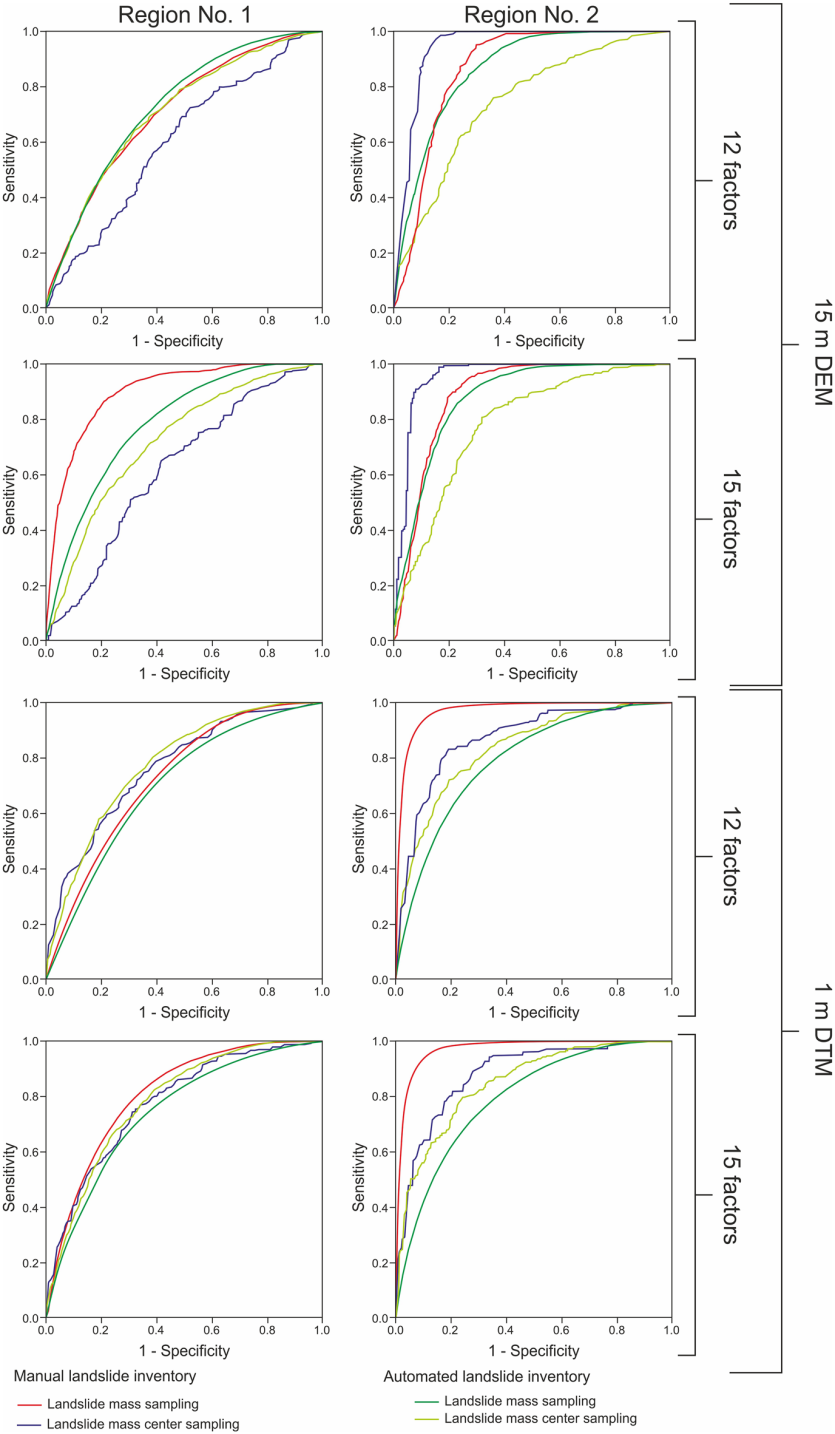
The receiver operation characteristic (ROC) curves for landslide susceptibility models for various input data. ROC curves were produced using IBM SPSS Statistics software (https://www.ibm.com/products/spss-statistics), and the final layout was created in CorelDRAW 2018 (https://www.coreldraw.com/en/).

We produced 32 landslide susceptibility models for two mountainous regions in SW Mexico using different input data (Fig. 4). Models differ in spatial resolution of topographic data used to extract landslide-causing factor values, the type of landslide inventory used to calibrate the coefficients of landslide-causing factors, several landslide-causing factors, and landslide sampling technique (Fig. 4). The goodness of fit and prediction accuracy of the developed susceptibility models are presented in Table S1, whereas the receiver operation characteristic (ROC) curves are presented in Fig. 5.

#### Region No. 1

##### 15 m resolution models

**Figure 6:**
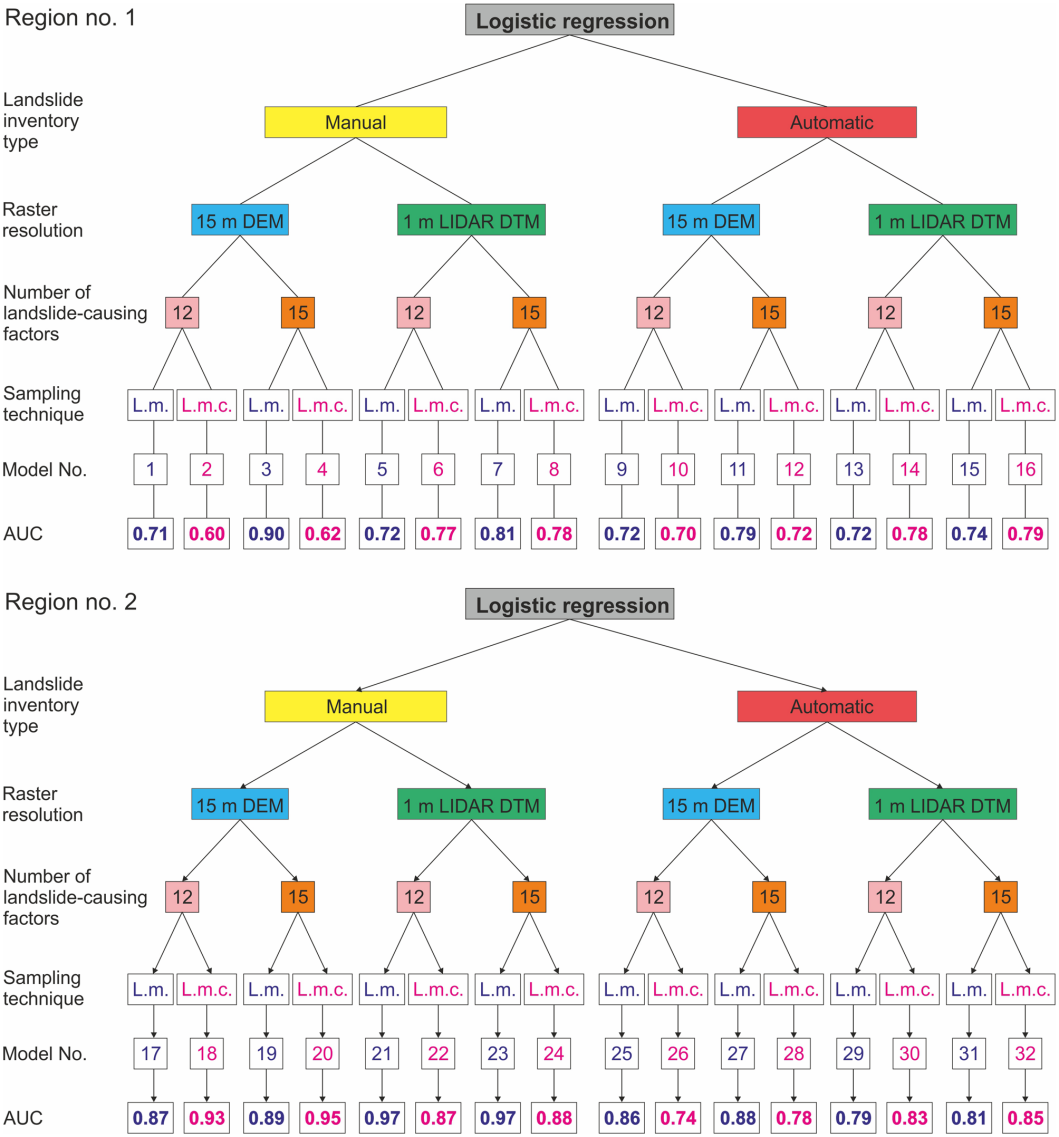
Comparison of the AUC (area under the curve) values for the resulting landslide susceptibility models based on different input data (see Table S2); landslide sampling technique: l.m.—landslide mass, l.m.c.—landslide mass center. Figure was designed and created in CorelDRAW 2018 (https://www.coreldraw.com/en/).

We produced 8 landslide susceptibility models for region No. 1 using 15 m resolution DEM data to extract landslide-causing factors (Table S1). All models show reasonable overall prediction accuracy values ranging significantly from 0.6 to 0.9 (Fig. 6 and Table S2). We calculated the highest AUC (area under the receiver operation characteristic curve) value (0.9) for model No. 5 based on the manual landslide inventory, including 15 landslide-causing factors and sampling data from landslide masses (Table S2). The lowest accuracy values were estimated for models No. 2 and 6 produced using manual inventory and sampling from landslide mass centers, 12 and 15 landslide causing factors (0.6–0.62; Fig. 6 and Table S2).

We found significant differences in the prediction accuracy of susceptibility models produced using a different number of landslide-causing factors, i.e., models produced using a higher number of landslide-causing factors show generally higher AUC values (up to 0.19 of the difference, except for model No. 6; Fig. 6 and Table S2), especially for sampling from landslide masses. We also observe the significant impact of the landslide sampling technique. Models produced using sampling from landslide mass show AUC values 0.11–0.18 higher than those generated using landslide mass centers for sampling. Variations in the sampling technique strongly affect the number of points used for the analysis, from < 40,000 points for sampling from the landslide mass to < 500 for sampling from the landslide mass center (Table S2).

In most cases, the susceptibility models derived from the automatic landslide inventory show higher overall prediction accuracy compared to the corresponding models based on manual inventory, using the same number of landslide-causing factors and landslide sampling technique (Fig. 6 and Table S2). We obtained a higher AUC value only for one model based on manual inventory, i.e., No. 5, using 15 landslide-causing factors and applying landslide mass for data sampling (Fig. 6).

##### 1 m resolution models

We produced 8 landslide susceptibility models using 1 m resolution LIDAR derived DTM that vary in input data type. All models show reasonable overall prediction accuracy, i.e., AUC values range from 0.72 to 0.81 (Fig. 6 and Table S2). We calculated the lowest prediction accuracy value (0.72) for two susceptibility models: (1) No. 9 based on manual event inventory using 12 landslide-causing factors and sampling from landslide mass; and (2) No. 11 based on automatic inventory, 12 landslide-causing factors, and sampling from landslide masses (Fig. 6 and Table S2). The highest accuracy was estimated for model No. 13 produced using the manual event inventory to extract 15 landslide-causing factors and sampling from landslide masses (Fig. 6 and Table S2). In all cases, the susceptibility models produced using 15 landslide-causing factors show higher prediction accuracy than those based on a lower number of causative agents. We found that models produced using landslide mass for sampling show higher prediction accuracy than those using landslide mass center. Overall prediction accuracy values are comparable for susceptibility models regardless of the type of landslide inventory used to calibrate the coefficients of the landslide-causing factors.

#### Region No. 2

##### 15 m resolution models

Susceptibility models for different input data based on the 15 m resolution DEM produced for region No. 2 and varying from 0.74 to 0.95 show higher AUC values than those calculated for region No. 1 (Fig. 6 and Table S2). The highest prediction accuracy (0.95) shows landslide susceptibility model No. 22 based on manual landslide inventory using 15 landslide-causing factors and sampling from the landslide mass center, whereas model No. 20 based on automatic landslide inventory, 12 landslide-causing factors, and sampling from landslide mass center present the lowest AUC value—0.74 (Fig. 6 and Table S2). Similarly, as for region No. 1, models produced using 15 landslide-causing factors present higher accuracy than those based on a lower number of causative agents. The impact of the landslide sampling technique is not that straightforward as for region No. 1. For manual landslide inventory, sampling from landslide mass center results in higher AUC values, whereas for models based on automatic inventory, higher accuracy is related to sampling from the landslide mass (Fig. 6 and Table S2).

Susceptibility models generated from automatic landslide inventory and sampling from landslide mass show high overall prediction accuracy (0.86–0.88), comparable with models based on manual inventory (Fig. 6 and Table S2). Only the models produced using sampling from the landslide mass center show lower prediction accuracy values (0.74–0.78; Fig. 6 and Table S2), which could be related to the insufficient number of points.

##### 1 m resolution models

Susceptibility models produced for region No. 2 based on 1 m resolution LIDAR derived DTM present the highest overall prediction accuracy, i.e., AUC values ranging from 0.79 to 0.97 (Fig. 6 and Table S2). We calculated the highest AUC value (0.97) for models No. 25 and 29, produced using manual inventory to extract landslide-causing factors and applying sampling from landslide masses. The highest accuracy was obtained regardless of the number of causative agents. On the other hand, the lowest accuracy value (0.79) was estimated for model No. 27 produced using the automatic landslide inventory, 12 landslide-causing factors, and sampling from landslide masses (Fig. 6 and Table S2). Generally, susceptibility models based on 15 landslide-causing factors show higher values of overall prediction accuracy than those using only 12 factors for logistic regression calculations. Moreover, we found that for models based on manual landslide inventory, sampling from landslide mass shows higher accuracy values, whereas, for a model produced using automatic inventory, the sampling from landslide mass centers results in higher AUC values (Table S1).

## Discussion

We produced 32 landslide susceptibility models with variable input data to assess the impact of variations in (1) the type of landslide inventory used to calibrate the coefficients of causative agents, (2) spatial resolution of topographic data used to extract landslide-causing factor values, (3) several landslide-causing factors, and (4) landslide sampling technique, on the resulting susceptibility maps (Figs. 4 and 6).

Differences in landslide susceptibility models produced using manual and automatic landslide inventories are insignificant, especially for the larger region No. 1 (Tables 2, 3, and S2). Even though AUC values for susceptibility models calibrated using landslides from the automatic inventory are usually lower than the corresponding models produced based on manually identified landslides, the difference is mainly less than 0.05 (Fig. 6 and Table S2). Lower accuracy of those models is related to the difference in objectives of the inventorying procedures, and hence with the landslide types captured by both inventories^15,19^. The visual interpretation of satellite images was used to detect landslides triggered by the rainfall related to Hurricane Manuel. Thus, this event inventory includes mainly debris flows that cluster in topographic convergence zones^15^. Regmi et al.^13^ showed that the accuracy of the susceptibility model produced by logistic regression would be higher if most of the landslides used in the analysis are caused or triggered by similar mechanisms. On the other hand, the CCM algorithm was employed to identify all past landslides features^15,32^. Hence, the inventory involves debris flows, mudflows, and deep-seated landslides that might occur in different slope conditions, convergence distance to streams, etc. Moreover, landslides triggered by different mechanisms, e.g., extreme precipitation events, earthquakes, human activity, might be included in this database as well. This results in susceptibility models of lower overall prediction accuracy. However, whereas models based on event inventory predict with high accuracy only landslides triggered by the same mechanism, e.g., extreme rainfall^15,20^, models generated from historical inventory could be used to forecast the location of any type of landslides, but with lower accuracy. The spatial distribution of different types of landslides strongly influenced the susceptibility model, which does not focus only on zones of topographic convergence, as in the case of debris flows from the manual inventory^42^, but also emphasis on the slope curvature, aspect, and distance to streams. Thus, the purpose of the study determines the selection of landslide inventory. In general, our results proved that susceptibility models based on automatic landslide inventory might present comparable accuracy as those generated from manual inventory.

**Table 2:** Mean values of landslide susceptibility models overall prediction accuracy (AUC) for different input data depending on the raster resolution of topographic data.

Input data		Region No. 1		Region No. 2	
		15 m DEM	1 m DTM	15 m DEM	1 m DTM
Number of landslide-causing factors	12 landslide-causing factors	0.68 ± 0.06	0.77 ± 0.04	0.85 ± 0.06	0.86 ± 0.07
	15 landslide-causing factors	0.76 ± 0.11	0.79 ± 0.03	0.88 ± 0.06	0.88 ± 0.06
Type of landslide inventory	Manual	0.71 ± 0.12	0.79 ± 0.04	0.89 ± 0.04	0.90 ± 0.06
	Automatic	0.73 ± 0.04	0.76 ± 0.03	0.82 ± 0.07	0.82 ± 0.03
Landslide sampling technique	Landslide mass	0.79 ± 0.09	0.77 ± 0.05	0.87 ± 0.02	0.87 ± 0.08
	Landslide mass center	0.65 ± 0.05	0.79 ± 0.02	0.86 ± 0.08	0.88 ± 0.04

Mean values with standard deviation.

**Table 3:** Mean values of landslide susceptibility models overall prediction accuracy (AUC) for different input data.

Input data		Region No. 1	Region No. 2
Number of landslide-causing factors	12 landslide-causing factors	0.72 ± 0.07	0.86 ± 0.06
	15 landslide-causing factors	0.78 ± 0.08	0.88 ± 0.06
Raster resolution of topographic data	15 m DEM	0.72 ± 0.09	0.86 ± 0.06
	1 m DTM	0.78 ± 0.04	0.88 ± 0.06
Type of landslide inventory	Manual	0.75 ± 0.09	0.89 ± 0.05
	Automatic	0.75 ± 0.04	0.82 ± 0.05
Landslide sampling technique	Landslide mass	0.78 ± 0.07	0.87 ± 0.06
	Landslide mass center	0.72 ± 0.08	0.87 ± 0.06

Mean values with standard deviation.

**Figure 7:**
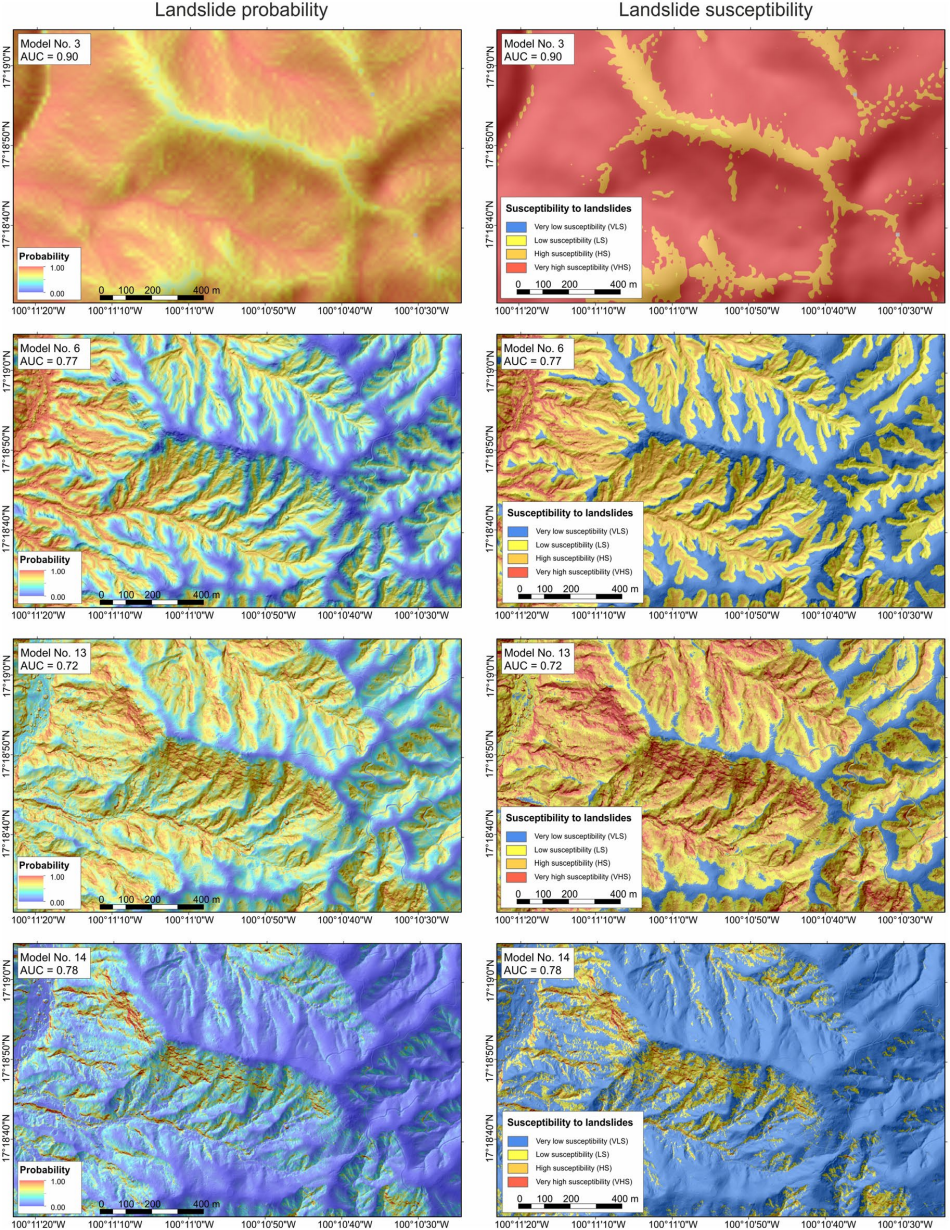
Comparison of selected landslide probability and susceptibility models produced for region No 1. Maps were generated using ArcMap 10.2.2 software, copyright and licensed by ESRI (http://desktop.arcgis.com/en/). The final layout was created in ArcMap 10.2.2 (http://desktop.arcgis.com/en/) and CorelDRAW 2018 (https://www.coreldraw.com/en/).

In general, landslide susceptibility models based on 1 m resolution topographic data show higher prediction accuracy than those derived from 15 m DEM (Tables 2, 3, and S2). Whereas AUC values obtained for the 15 m models range from 0.6 to 0.9 (mean values: 0.72 ± 0.09 for region No. 1 and 0.86 ± 0.06 for region No. 2), those for 1 m start at the level of 0.72 and reach up to 0.97 (mean values: 0.78 ± 0.04 for region No. 1 and 0.88 ± 0.06 for region No. 2; Tables S2 and 3). However, in many cases, the difference is inconsiderable, or even the AUC values are higher for models developed using 15 m resolution data (Fig. 6 and Table S2). This could be related to a smoothing effect on landscape topographical representation^21^. The smoothing effect results in a larger area being included in high and very high susceptibility zones for lower resolution data. Tian et al.^22^, Mahalingan and Olsen^23^, and Chang et al.^43^ showed that a higher resolution of topographic data does not necessarily lead to susceptibility maps of higher accuracy. However, in contrast to Tian et al.^22^, we found that the landslide susceptibility mapping is more sensitive to the resolution of topographic data in areas of high relative relief. Higher-resolution DTMs might introduce more noise than the lower resolution models, but unlike Chang et al.^43^, we found that after proper data processing, and especially using automatic landslide inventories, these models perform the same if not better (Fig. 6 and Tables S1, S2). On the other hand, the value of overall prediction accuracy cannot be the only parameter indicating appropriate susceptibility models. For example, models produced using topographic data in coarse resolution usually show relatively high accuracy related to large areas of high and very high landslide probability (see also^9,10,13,14,22,44^). Whereas models based on LIDAR data, even when showing lower accuracy, present in detailed scales the variations of landslide probability and susceptibility along and across slopes, and thus can be more meaningful from a geomorphological perspective (Fig. 7)^4,15^. Thus, high-resolution LIDAR topographic data indeed improve landslide susceptibility mapping, enhancing precision in identifying high and very high susceptibility zones.

Our results suggest that the importance of landslide-causing factors depends on the resolution of topographic data, i.e., low-resolution topographic data requires a higher number of causative factors (Tables 2 and 3). The mean differences between AUC values obtained for models based on 15 and 12 landslide-causing factors reach up to 0.08 for low-resolution topographic data, and only 0.02 for high-resolution LIDAR derived (Table 2). Thus, if high-resolution data is used, even fewer independent landslide-causing factors can result in satisfactory maps with high predictive capability, i.e., AUC > 0.7 (0.77 ± 0.04 for region No. 1 and 0.86 ± 0.07 for region No. 2; Tables 2, 3 and S2). This agrees with Mahalingam et al.^45^, who also showed that regardless of the applied landslide susceptibility method when LIDAR data is used, even a few factors could lead to models with reasonable accuracy.

The impact of landslide-causing factor number appears to be contingent on the landslide inventory type and is more notable for models based on manual inventory (Fig. 6 and Table S2). This suggests that for automatic inventory, topographic and hydrographic factors extracted directly from the LIDAR-derived DTM are sufficient to obtain models with reasonable accuracy. Additional data, such as vegetation or distance to roads and paths, improve the resulting models only inconsiderable. Thus, the landslide susceptibility model can be made entirely automatically using the CCM algorithm and logistic regression method, but the LIDAR data is required to obtain high prediction accuracy.

Our results suggest that sampling from landslide masses is more appropriate for mapping landslide susceptibility than sampling from landslide mass centers (Fig. 6 and Tables 2, 3, and S2). Similar results were obtained by Regmi et al.^13^. This approach produces more reliable models with higher or similar overall prediction accuracy (e.g., 0.78 ± 0.07 versus 0.72 ± 0.08 for region No. 1; Table 3). Whereas sampling from landslide masses results in an extensive landslide and non-landslide point population (> 8,000,000 points for region No. 1; Table S1), data from landslide mass centers leads to a limited number of points in the database (e.g., only 354 in the case of the manual landslide inventory in the region No. 2; Table S1). Susceptibility models based on fewer data might result in high but false nonsignificant prediction accuracy, which is proven by visual inspection of resulting susceptibility models. Moreover, the results of logistic regression analysis calculated for low datasets are questionable. Moreover, only one point for each landslide feature equalizes small, insignificant shallow landslides with large deep-seated features, and using only one single value of a landslide-causing factor might be misleading. Thus, sampling from the centers of the landslide masses enhances the uncertainty of the resulting susceptibility models.

## Conclusions

To assess the influence of the input data on landslide susceptibility mapping, we produced 32 landslide susceptibility models for various input data sets. The obtained results can be summarized in the following conclusions:

- Variations in precision and accuracy of susceptibility models produced using different landslide databases, including manual and automatic inventories, are insignificant. Observed small differences are related to the contrasting objectives of the inventorying procedures and thus to variations in landslide type and number captured by both inventories. Nevertheless, the comparable overall prediction accuracy values prove the applicability of the entirely automated process of landslide susceptibility mapping.
- Susceptibility models based on 1 m resolution LIDAR derived DTMs are more precise and show higher prediction accuracy than those developed using 15 m resolution DEM. Visual inspection of these models reveals that these present in detailed scales the variations of landslide probability and susceptibility along and across slopes and would be more appropriate for urban planning, land management, and safe human occupation.
- The substantial impact of landslide-causing factors is observed in models based on manual landslide inventory, i.e., the higher the number of causative agents used in logistic regression analysis, the more accurate the landslide susceptibility model.
- The influence of the number of landslide-causing factors is insignificant for automatic landslide inventory and high-resolution topographic data, i.e., even the lower number of causative agents results in highly accurate susceptibility maps.
- Sampling from landslide masses is more appropriate for mapping landslide susceptibility than sampling from the landslide mass center, as the small datasets produced by the landslide mass center technique might lead to high but false nonsignificant prediction accuracy.

Overall, to map landslide susceptibility for each study case, the most appropriate input data (e.g., landslide inventory type, raster resolution of topographic data, number of landslide-causing factors) and techniques (i.e., data sampling method) need to be selected after a detailed assessment of the input data, their quality, and resolution, as well as the purpose of the susceptibility mapping. This study demonstrated that most produced landslide susceptibility models, even though variable, present reasonable overall prediction accuracy.

## Methods and materials

### Topographic data

To investigate the effect of DEM resolution on landslide susceptibility mapping, we used topographic data in two different grid sizes: 1) 15 m resolution digital elevation model (DEM), and 2) 1 m resolution LIDAR derived digital terrain model (DTM; Fig. 4). The 15 m DEM with 4.8 m means squared error was acquired from The National Institute of Statistics and Geography (INEGI*, Instituto Nacional de Estadistica y Geografia*; http://www.inegi.org.mx/). The model was generated using Geodetic Reference System 1980 (GRS 80), The International Terrestrial Reference Frame 1992 (ITRF92) epoch 1988.0, and geographical coordinates.

The LIDAR topographic data with a nominal density of 8 points/m^2^ and altimetric accuracy of 35 cm was gathered on 19 March 2015 using a CESSNA TU206H aircraft flying at the elevation of 700 m above ground. The data was collected by a RIEGL Q-780 airborne laser scanner with a laser pulse repetition rate of 400 Hz and field of view (FOV) of 60° (+ 30° /—30°). RIEGL software was used to process the data. The resulted "LAS" point cloud was classified into ground and default points (e.g., vegetation, infrastructure, buildings, etc.) using TerraScan and TerraModeler (https://www.terrasolid.com/home.php), followed by manual verification. Finally, we produced a 1 m resolution raster version of the DTM from sampling the Triangulated Irregular Network (TIN) generated for the bare earth ground classified point cloud data.

### Landslide inventory

This study used two different landslide inventories, i.e., (a) manual and (b) automatic (Fig. 4). The first one was produced using manual identification of landslide features based on satellite images captured before and after Hurricane Manuel that hit western Mexico in September 2013, i.e., 2011 (7 March 2011) and 2014 (13 April 2014, 16 April 2014, and 12 May 2014) (Image © 2016 DigitalGlobe). It is an event inventory, as it presents landslides caused by a single trigger^19^. To produce automatic landslide inventory, we applied the automated Contour Connection Method (CCM) using LIDAR data^15,32^. The algorithm employed in this method uses the shape of topographic features to delineate past landslides. For that, the LIDAR DTM was first smoothed with focal statistics by averaging elevations within a 3 × 3 window in the ArcMap 10.2.2 software (https://desktop.arcgis.com/en/arcmap). This is a historical inventory as it shows the cumulative effects of many landslide events with no information on the age of identified features^19^. The accuracy of both inventories was assessed by visually comparing them with mapped and verified in the field landslide extends (i.e., reference data) in a selected 1 km^2^ test area. We also calculated the overall accuracy following the procedure in Zhan et al.^46^ and Al-Rawabdeh et al.^41^. For the validation of manual inventory, we used only landslides produced by Hurricane Manuel. In contrast, for the automatic inventory, we utilized all identified features.

### Logistic regression method

We used the logistic regression method to produce maps of landslide probability and susceptibility for different input data. The applicability of this approach in mapping landslide susceptibility with reasonable accuracy compared to other probabilistic methods has been shown^12,13^. In this study, we followed the procedure described already in literature^9,10,12–15,44,47^. The logistic regression analysis was conducted using the IBM SPSS Statistics software (https://www.ibm.com/products/spss-statistics), whereas input data preparation, landslide-causing factors calculations, final map computations and visualizations were performed using the ArcMap 10.2.2 software with Python Scripting (https://desktop.arcgis.com/en/arcmap). The final susceptibility map was produced as a classified probability of the landslide occurrence, which varies from 0 (no susceptibility) to 1 (complete susceptibility). We grouped landslide susceptibility into four equal intervals e.g.,^9,10,15^: 0–0.25 very low susceptibility, 0.25–0.5 low susceptibility, 0.5–0.75 high susceptibility, and 0.75–1 very high susceptibility.

### Validity analysis

To test the validity of the presented landslide susceptibility analyses, we followed the procedure proposed by Regmi et al.^13^ and Gaidzik et al.^15^. In each model, half of the landslide inventory was used for training the statistical models, while the remaining 50% was used for validation purposes (testing datasets). Thus, the susceptibility models produced using the training manual landslide inventory were subsequently validated using the testing data from the remaining part of this inventory. The same applies to models based on the automatic landslide inventory. Because both inventories present different features and time frames, we did not compare the resulting susceptibility maps with a different type of inventory, as the results might be misleading and obliterate the main purpose of this study. The results of such an alternating comparison can be found in Gaidzik et al.^15^.

The method's performance was evaluated by the receiver operation characteristic (ROC) curve and the area under the curve (AUC). This is a commonly used method for assessing the overall accuracy of landslide susceptibility mapping e.g.,^4,13,15^. The curve is a plot of the probability of a correctly predicted landslide occurrence versus the probability of a falsely predicted landslide occurrence (i.e., a prediction of a landslide for a location where a landslide did not occur^13^.

### Input data

We produced numerous susceptibility models to assess the effect of different input data on the accuracy of landslide susceptibility mapping (Fig. 4). We used 15 resolution DEM, and 1 m resolution LIDAR derived DTM to extract landslide-causing factors (Fig. 4). We applied 12 landslide-causing factors directly extracted from topographic data that were proven to produce landslide susceptibility models with reasonable prediction accuracy^15^: elevation, slope, aspect, tangential curvature, plan curvature, profile curvature, flow length, flow accumulation, topographic wetness index (*TWI*), stream power index (*SPI*), solar radiation and distance to stream. However, to evaluate the effect of additional landslide-causing factors not related to topography, we also incorporated vegetation and distance to main roads and distance to paths (Fig. 4). These three further factors were chosen based on their crucial importance, apart from the extreme precipitation, in the development of the La Pintada landslide that occurred in this region in 2013, and resulted in 71 fatalities, leading to its ranking as one of the deadliest landslides in Mexico^40^. We did not include data on lithology, land use, and soil that could also influence landslide formation^13^ because for the study area, these data sets lack the resolution to provide useful landslide susceptibility information and do not show any differentiation. For the calibration of landslide-causing factor coefficients, we applied two different landslide inventory databases: manual and automatic (Fig. 4). Because of the predominance of debris flows and a limited number of other landslide types, we used only an unclassified database (i.e., all identified landslides). For landslide sampling, we used (1) samples from the centers of the landslide masses and an equal number of random samples from areas free of landslides e.g., ^13,15^, and (2) samples from each cell of the landslide mass (entire area of a landslide) and an equal number of random samples from areas free of landslides e.g., ^13^ (Fig. 4). Thus, there is only one point for each identified landslide in the first method, regardless of its size. In the second one—the larger the landslide, the more sampling points used in the modeling, i.e., the input of large features, i.e., high magnitude^19^, is more significant in the final results. We used the same number of landslide and non-landslide cells to extract data from maps of landslide-causing factors to eliminate bias in the sampling process. We used the same amount of landslide and non-landslide cells to extract data from maps of landslide-causing factors to eliminate bias in the sampling process.

## Supplementary Information

Supplementary Information.

## Data Availability

The datasets generated during and analysed during the current study are available from the corresponding author on reasonable request.
