# Supplementary material for: The importance of input data on landslide susceptibility mapping
Source: Sci Rep. 2021 Sep 29;11:19334. doi: 10.1038/s41598-021-98830-y (PMC8481530; doi:10.1038/s41598-021-98830-y)
Supplement: Supplementary file 1 — Supplementary Information. [file 41598_2021_98830_MOESM1_ESM.pdf]

The importance of input data on landslide susceptibility mapping

Krzysztof Gaidzik[1*], María Teresa Ramírez-Herrera[2]

[1]Institute of Earth Sciences, University of Silesia, Będzińska 60, 41-200 Sosnowiec, Poland

[2]Laboratorio de Tsunamis y Paleosismología, Instituto de Geografía, Universidad Nacional Autónoma de México, Ciudad Universitaria, Coyoacán, 04510 México, Ciudad de México, México. .

[*]Corresponding author: krzysztof.gaidzik@us.edu.pl

Supplementary materials

Table S1. Statistics of the logistic regression applied to produce maps of landslide probability and susceptibility for different input data.

Table S2. Overall prediction accuracy (area under the Curve, AUC) for 32 susceptibility models for different input data (see Figure 6).

Table S1. Statistics of the logistic regression applied to produce maps of landslide probability and susceptibility for different input data.

Region	Raster resolution	No. of landslide-causing factors	Landslide inventory type	Sampling technique	Model No.	Number of sampling cells		Likelihood ratio test		Chi-square	p	Cox and Snell R^2	Nagelkerke R^2
						Landslide occurrence	Landslide absence	$-2 \ln L_0$	$-2 \ln L$				
Region No. 1	15 m DEM	12	Manual	Landslide mass	1	2,550	2,550	6,703.36	6,369.09	19.32	0.01	0.13	0.17
				Landslide mass center	2	243	243	657.98	652.36	8.85	0.36	0.32	0.43
			Automatic	Landslide mass	3	18,522	18,522	48,589.24	45,348.41	266.50	0.00	0.15	0.20
				Landslide mass center	4	1,382	1,382	3,681.25	3,476.74	10.26	0.25	0.12	0.16
		15	Manual	Landslide mass	5	2,550	2,550	6,249.33	4,115.50	93.50	0.00	0.44	0.59
				Landslide mass center	6	243	243	666.48	651.08	12.06	0.15	0.05	0.06
			Automatic	Landslide mass	7	18,522	18,522	48,589.24	41,116.26	147.67	0.00	0.25	0.32
				Landslide mass center	8	1,382	1,382	3,681.25	3,417.04	17.06	0.03	0.14	0.19
	1 m DTM	12	Manual	Landslide mass	9	511,780	511,780	1,267,388.79	1,221,132.33	2,619.01	0.00	0.18	0.23
				Landslide mass center	10	243	243	563.66	562.24	16.16	0.04	0.21	0.27
			Automatic	Landslide mass	11	4,147,525	4,147,525	10,682,380.6	10,117,854.7	45,989.26	0.00	0.15	0.21
				Landslide mass center	12	1,382	1,382	3,139.02	3,108.11	17.59	0.03	0.23	0.31
		15	Manual	Landslide mass	13	511,780	511,780	1,336,902.24	1,140,017.42	5,647.78	0.00	0.28	0.37
				Landslide mass center	14	243	243	603.49	569.87	1.60	0.99	0.19	0.26

			Automatic	Landslide mass	15	4,147,525	4,147,525	10,682,380.6	9,917,807.1	32.769.79	0.00	0.17	0.23
				Landslide mass center	16	1,382	1,382	3,471.17	3,083.57	18.38	0,02	0.24	0.32
Region No. 2	15 m DEM	12	Manual	Landslide mass	17	761	761	1,769.49	1,316.28	112.10	0.00	0.41	0.54
				Landslide mass center	18	177	177	362.61	221.88	10.11	0.26	0.53	0.71
			Automatic	Landslide mass	19	3,843	3,843	9,285.63	6,973.30	48.50	0.00	0.38	0.51
				Landslide mass center	20	344	344	991.56	836.09	9.83	0.28	0.16	0.21
		15	Manual	Landslide mass	21	761	761	1,374.44	1,167.25	33.17	0.00	0.46	0.62
				Landslide mass center	22	177	177	274.07	167.53	7.40	0.49	0.60	0.80
			Automatic	Landslide mass	23	3,843	3,843	8,353.95	6,489.62	82.83	0.00	0.42	0.56
				Landslide mass center	24	344	344	902.84	795.48	14.62	0.07	0.21	0.27
	1 m DTM	12	Manual	Landslide mass	25	136,575	136,575	338.28	230.10	29.03	0.00	0.42	0.56
				Landslide mass center	26	177	177	374.01	320.37	9.32	0.32	0.38	0.51
			Automatic	Landslide mass	27	863,927	863,927	2,072,790.78	1,921,381.86	9,336.14	0.00	0.24	0.32
				Landslide mass center	28	344	344	785.00	697.00	4.90	0.77	0.31	0.42
		15	Manual	Landslide mass	29	136,575	136,575	338,276.14	227,935.64	26,693.31	0.00	0.42	0.57
				Landslide mass center	30	177	177	307.07	298.91	9.34	0.32	0.42	0.55
			Automatic	Landslide mass	31	863,927	863,927	2,072,790.78	1,851,596.92	6,231.04	0.00	0.27	0.36
				Landslide mass center	32	344	344	785.00	657.97	4.16	0.84	0.35	0.47

Table S2. Overall prediction accuracy (area under the Curve, AUC) for 32 susceptibility models for different input data (see Figure 6).

	Raster resolution	Number of landslide-causing factors	Inventory type	Sampling technique	Model no.	AUC
Region No. 1	15 m DEM	12	Manual	Landslide mass	1	0.71
				Landslide mass center	2	0.60
			Automatic	Landslide mass	3	0.72
				Landslide mass center	4	0.70
		15	Manual	Landslide mass	5	0.90
				Landslide mass center	6	0.62
			Automatic	Landslide mass	7	0.79
				Landslide mass center	8	0.72
	1 m DTM	12	Manual	Landslide mass	9	0.72
				Landslide mass center	10	0.77
			Automatic	Landslide mass	11	0.72
				Landslide mass center	12	0.78
		15	Manual	Landslide mass	13	0.81
				Landslide mass center	14	0.78
			Automatic	Landslide mass	15	0.74
				Landslide mass center	16	0.79
Region No. 2	15 m DEM	12	Manual	Landslide mass	17	0.87
				Landslide mass center	18	0.93
			Automatic	Landslide mass	19	0.86
				Landslide mass center	20	0.74
		15	Manual	Landslide mass	21	0.89
				Landslide mass center	22	0.95
			Automatic	Landslide mass	23	0.88
				Landslide mass center	24	0.78
	1 m DTM	12	Manual	Landslide mass	25	0.97
				Landslide mass center	26	0.87
			Automatic	Landslide mass	27	0.79
				Landslide mass center	28	0.83
		15	Manual	Landslide mass	29	0.97
				Landslide mass center	30	0.88
			Automatic	Landslide mass	31	0.81
				Landslide mass center	32	0.85
